# Supplementary material for: Barnacles as biological flow indicators
Source: PeerJ. 2023 Apr 18;11:e15018. doi: 10.7717/peerj.15018 (PMC10120587; doi:10.7717/peerj.15018)
Supplement: Supplemental Information 1 — Map of Texas produced in R:”maps”. Google Earth image of field sites for experiment 1 (Google, ©2016). [file peerj-11-15018-s001.pdf]

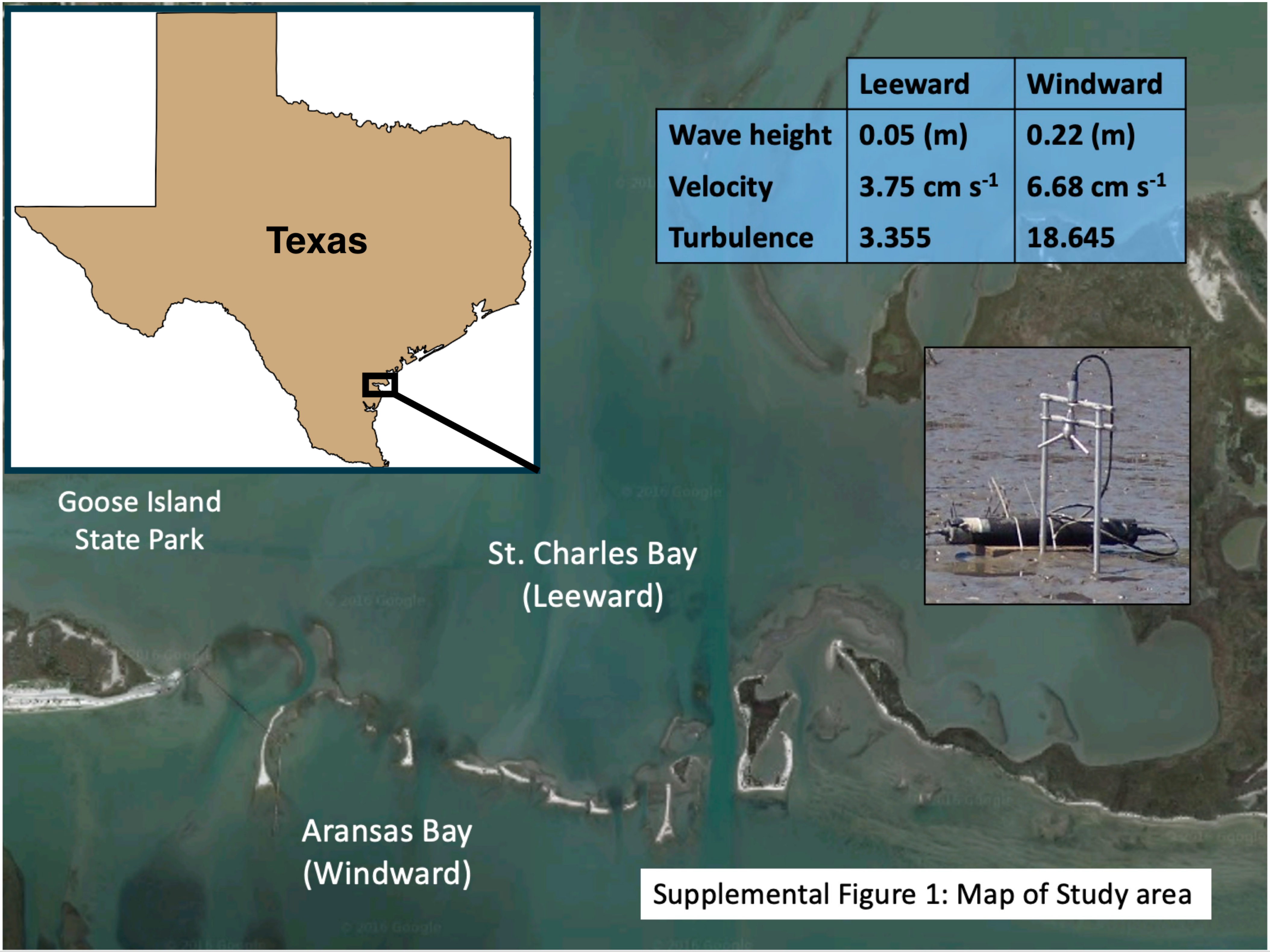

	Leeward	Windward
Wave height	0.05 (m)	0.22 (m)
Velocity	3.75 cm s^{-1}	6.68 cm s^{-1}
Turbulence	3.355	18.645

Supplemental Figure 1: Map of Study area
