# Supplementary figures and images for: Barnacles as biological flow indicators
Source: PeerJ. 2023 Apr 18;11:e15018. doi: 10.7717/peerj.15018 (PMC10120587; doi:10.7717/peerj.15018)

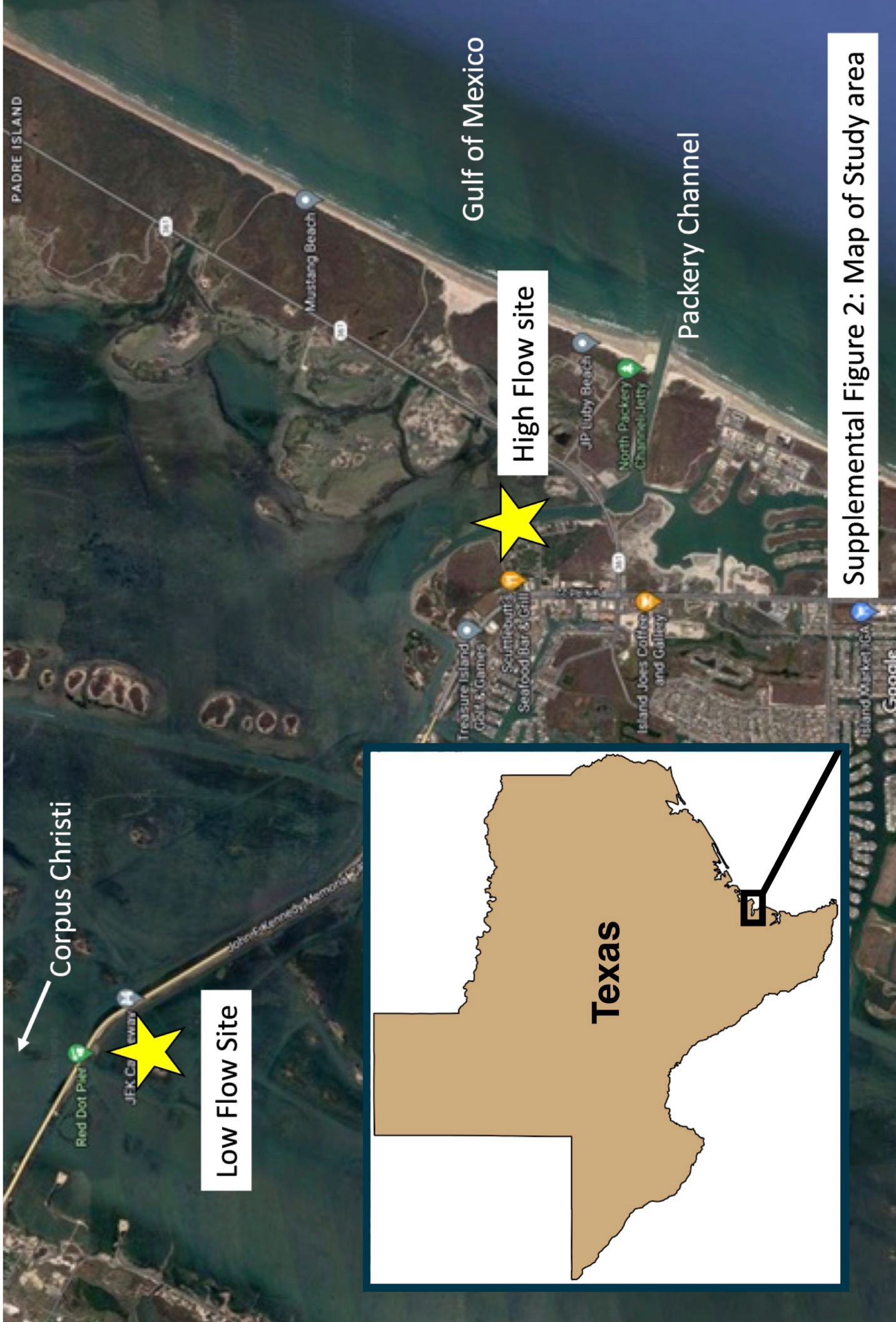

Supplemental Figure 2: Map of Study area

Supplement: Supplemental Information 2 — Map of Texas produced in R:”maps”. Google Earth image of field sites for experiment 2 (Google, ©2022). [file peerj-11-15018-s002.pdf]
