# Supplementary material for: Barnacles as biological flow indicators
Source: PeerJ. 2023 Apr 18;11:e15018. doi: 10.7717/peerj.15018 (PMC10120587; doi:10.7717/peerj.15018)
Supplement: Supplemental Information 3 — Mass loss of Life Savers™ in different flow velocities when protected by a shield or unprotected (control). Significantly less mass loss occurred behind the shield than in controls, indicating the shield effectively reduced flow velocity. Each data point is the mean mass loss of 5 Life Savers™. [file peerj-11-15018-s003.pdf]

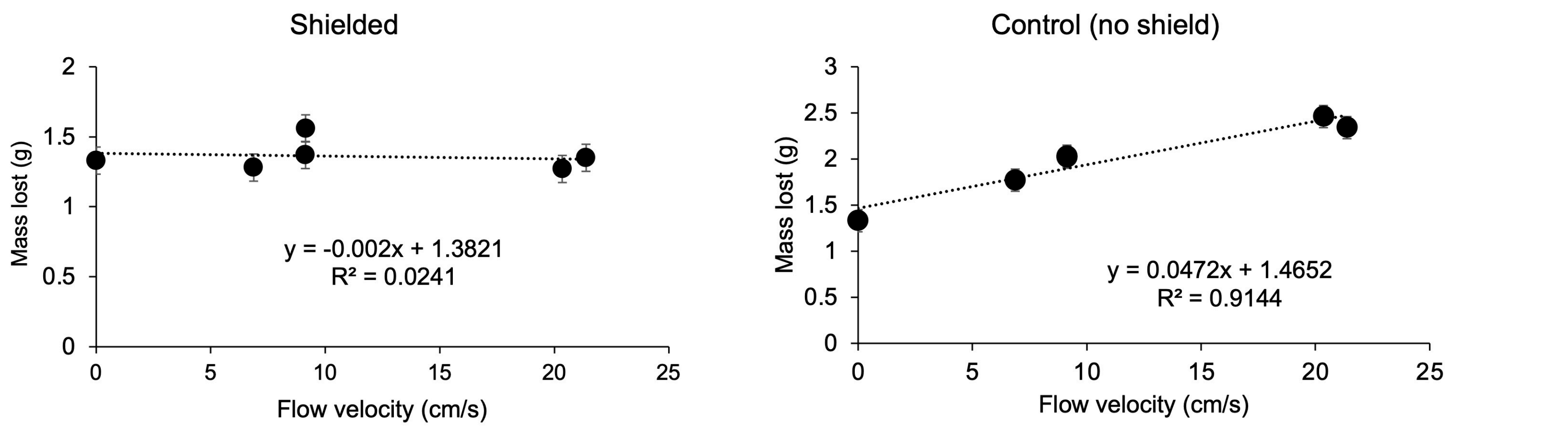

Supplemental Figure 3: Mass loss of Life Savers ™ in different flow velocities when protected by a shield or unprotected (control). Significantly less mass loss occurred behind the shield than in controls, indicating the shield effectively reduced flow velocity. Each data point is the mean mass loss of 5 Life Savers ™.
